# Editorial: Invisible hazards, lasting impact: airborne toxicants and systemic disease from environmental and occupational exposures

**DOI:** 10.3389/fpubh.2026.1943584

**Published:** 2026-09-10

**Authors:** Tammy A. Butterick, Julie M. Tomáška, Pamala Ginex, Janeen H. Trembley, Paul Barach

**Affiliations:** 1 Minneapolis Veterans Affairs Health Care System, Minneapolis, MN, United States; 2 Center for Veterans Research and Education, Minneapolis, MN, United States; 3 Department of Neuroscience, University of Minnesota, Minneapolis, MN, United States; 4 Department of Food Science and Nutrition, University of Minnesota, St. Paul, MN, United States; 5 Burn Pits 360 Veterans Organization, Robstown, TX, United States; 6 School of Nursing, Stony Brook University, Stony Brook, NY, United States; 7 Department of Laboratory Medicine and Pathology, University of Minnesota, Minneapolis, MN, United States; 8 Masonic Cancer Center, University of Minnesota, Minneapolis, MN, United States; 9 College of Population Health, Thomas Jefferson University, Philadelphia, PA, United States; 10 Sheps Health Services Research Center, University of North Carolina, Chapel Hill, NC, United States

**Keywords:** military burn pits, environmental toxicology, exposome, fire fighters, precision public health, systems science, Veterans health, wildfire smoke

## From challenges to convergence opportunities

Everyday millions of people inhale invisible hazards whose biological consequences may not become apparent for decades. Yet these exposures rarely occur in isolation. They arise from complex interactions among work, environment, technology, policy, climate, and social systems. Understanding and preventing their effects therefore requires moving beyond toxicology toward systems thinking (1–3). The studies assembled in this Research Topic demonstrate that environmental and occupational exposures remain among the largest yet least visible drivers of preventable morbidity and mortality worldwide with long latency after exposure, creating substantial challenges for disease recognition, surveillance, prevention, and healthcare delivery (4). The investigations presented in this Research Topic span molecular, microbiologic, clinical, epidemiologic, and occupational sciences and collectively demonstrate that inhaled toxicants rarely remain confined to the respiratory tract but instead contribute to interconnected biological processes affecting multiple organ systems, challenging traditional single-agent and organ-specific paradigms of environmental health research (5, 6).

The experiences of United States (U.S.) military Veterans exposed to environmental toxicants illustrate these challenges particularly well. Environmental exposures have

accompanied warfare throughout history, yet it has often taken decades for affected Veterans to receive recognition, healthcare services, and scientific explanations for their illnesses (7–9). The delayed recognition of diseases associated with Agent Orange, Gulf War exposures, and burn pit exposures reflects not only the biological complexity of environmental toxicants, but also the limitations of fragmented scientific, healthcare, surveillance, and policy systems (10, 11). In this context, Veterans, firefighters, miners, disaster responders and healthcare workers serve as important sentinel populations for understanding environmental health risks more broadly, providing opportunities to investigate mechanisms of injury, disease latency, biomarkers of exposure, and long-term health outcomes (9, 12–14). Lessons learned from these populations may ultimately inform responses to other emerging environmental threats, including wildfire smoke, industrial disasters, urban pollution, and climate-associated exposures (15). Public trust, transparent communication, and community engagement are increasingly recognized as essential components of effective environmental surveillance and policy implementation.

Taken together, the studies presented in this Research Topic support a new paradigm for environmental and occupational health research that prioritizes convergence science and integration across the physical, biological, clinical, and population sciences (3, 16, 17). Advancing environmental health will require not only scientific discovery, but also new models of collaboration capable of transforming fragmented knowledge into effective surveillance, prevention, healthcare delivery, and public policy.

## Airborne toxicants as drivers of multisystem disease

A major theme emerging from this Research Topic is that airborne toxicants exert effects far beyond the respiratory tract. Zhang et al., demonstrated that even low levels of PM_2_.5 exposure were associated with increased emergency department visits for cardiovascular disease, reinforcing evidence that adverse health effects may occur even at relatively low exposure levels. Collectively the studies by Xue et al., Yu et al., Du et al., Wen et al., and Glick et al. further highlight the complex relationships among environmental exposures, inflammation, allergic disease, metabolic dysfunction, cardiovascular injury, and chronic systemic illness well beyond the respiratory tract.

Real-world exposures occur as complex mixtures of particulate matter, metals, allergens, combustion products, volatile organic compounds, and emerging contaminants that interact across biological systems and throughout the life course (2, 18, 19). Studies examining combined environmental exposures, multiple chemical sensitivity, and emerging contaminants such as microplastics emphasize the need to move beyond single-contaminant frameworks (20, 21). Together, these findings support the emerging exposome framework, which recognizes that cumulative environmental exposures interact with biological, occupational, and behavioral factors to shape disease risk and progression (1, 2, 19).

## Military Veterans as sentinel populations for complex exposure biology

Military Veterans represent a pivotal sentinel population for understanding the long-term consequences of complex inhalational exposures (7–9). Service members deployed to combat environments encountered unique combinations of burn pit emissions, combustion products, desert dust, blast overpressure, and other environmental hazards under conditions rarely experienced by civilian populations.

Studies in this Research Topic provide critical insights into deployment-related health outcomes and mechanisms of disease. Investigations of post-9/11 Veterans with blast exposure by Glick et al. identified persistent respiratory symptoms and altered pulmonary physiology, while experimental studies by Frey et al. demonstrated that burn pit emissions and sand inhalation alter the lung microbiome. Studies by Teitz-Tennenbaum et al. identified immune activation, inflammatory signaling, and tissue remodeling pathways associated with deployment-related constrictive bronchiolitis, and findings by Connick et al. further support the concept that environmental exposures induce persistent biological changes contributing to multisystem disease.

Military populations provide a unique natural experiment in complex exposure biology (9). These observations underscore the importance of integrating injury epidemiology with exposure science, environmental toxicology, clinical medicine, and public health to better characterize exposure-disease relationships, identify sentinel events, strengthen surveillance systems, and improve prevention and healthcare delivery (3, 9, 15, 17). Lessons learned from military populations may ultimately inform responses to broader environmental and occupational health challenges (9, 22).

## Exposure science as systems science

A major conceptual advance emerging from this Research Topic is the recognition that environmental and occupational exposures should increasingly be viewed through the lens of safety science and systems thinking (17, 23). Much as patient safety has evolved from blaming individual clinicians toward understanding latent organizational failures, environmental disease prevention requires moving beyond individual behavior toward identifying weaknesses in the design of workplaces, technologies, regulatory systems, and organizational culture that shape exposure risk (24).

Generating evidence alone rarely changes practice. Successful exposure prevention depends upon implementation strategies that facilitate adoption, sustainability, stakeholder engagement, organizational readiness, continuous evaluation, and policy translation. Similar to adverse events in healthcare and other high-risk industries, exposure-related disease often emerges through interactions among multiple system-level factors rather than from a single hazard or failure (24, 25). Generating evidence alone rarely changes practice. Successful exposure prevention depends upon implementation strategies that facilitate adoption, sustainability, stakeholder engagement, organizational readiness, continuous evaluation, and policy translation (16, 26, 27).

Studies by Genovese et al., Caban-Martinez et al., and Rumi et al. illustrate the value of moving beyond traditional hazard identification toward integrated approaches incorporating exposure surveillance, risk anticipation, organizational learning, and resilience-based prevention strategies. Applying systems-oriented approaches to environmental and occupational health may improve exposure characterization, facilitate earlier disease recognition, strengthen prevention strategies, and inform more effective public health and policy interventions (3, 23).

## First responders, healthcare workers, climate change, and emerging exposure threats

The contributions within this Research Topic highlight how rapidly evolving environmental conditions are creating increasingly complex exposure challenges. Studies by Caban-Martinez et al. and Penuelas et al. underscore the occupational risks faced by firefighters and fire investigators, while work by demonstrates how climate change, urbanization, and changing built environments are reshaping environmental exposure landscapes. First responders, healthcare workers, emergency personnel, and disaster response teams occupy a uniquely vulnerable position at the intersection of occupational health and environmental disaster response and may serve as essential sentinel populations for understanding the long-term consequences of repeated environmental exposures (13, 22, 28). Protecting these populations will require advances in exposure assessment, surveillance, disaster preparedness, occupational health systems, injury epidemiology, and resilience science (22, 23, 29).

## Toward systems-based environmental health

**Figure 1:**
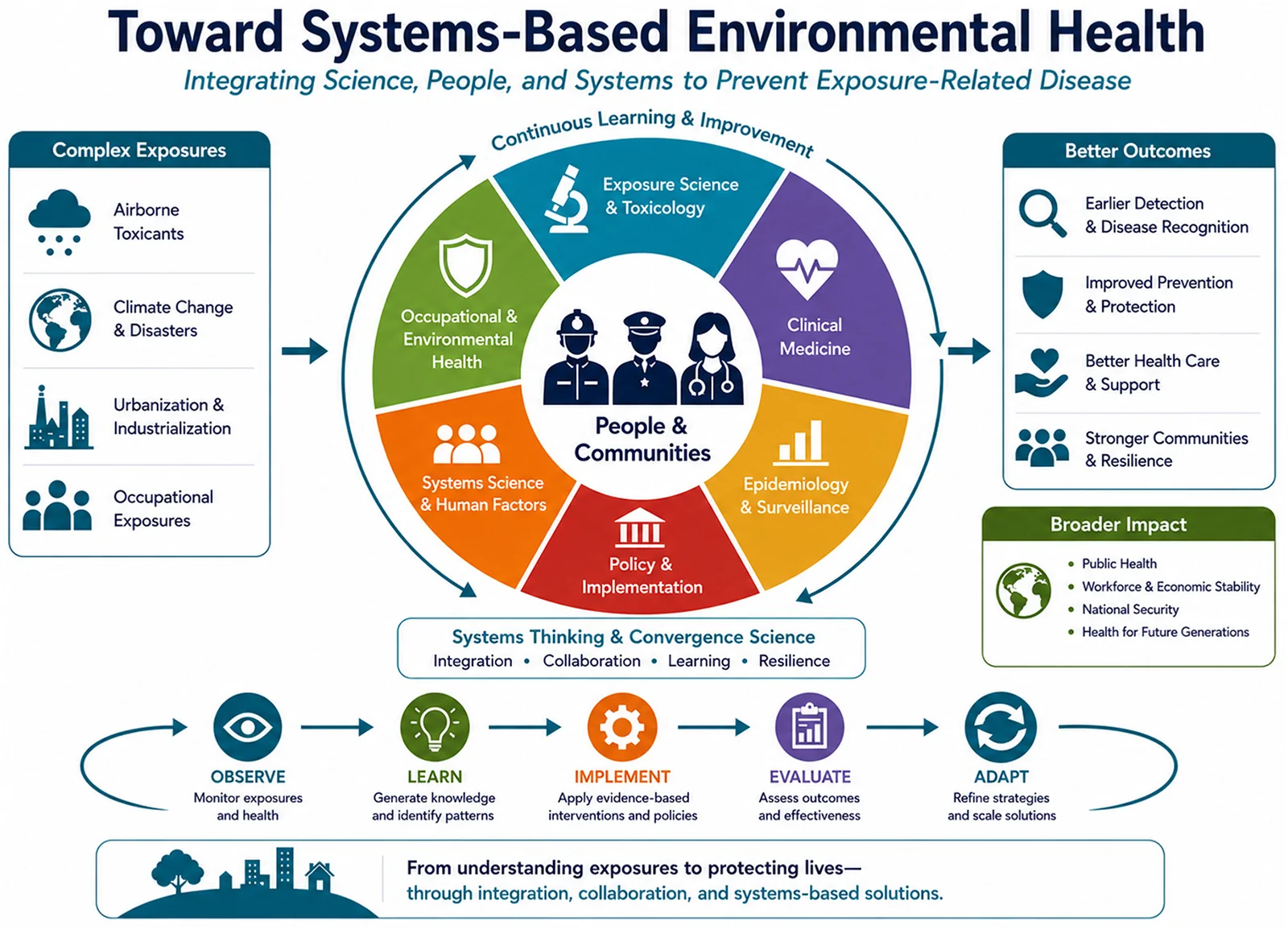
Toward systems-based environmental health. Conceptual framework illustrating how environmental and occupational exposures interact with biological, occupational, environmental, and societal systems to influence health outcomes. The figure highlights the integration of exposure science, toxicology, clinical medicine, epidemiology, occupational and environmental health, systems science, human factors, and policy to support interdisciplinary collaboration, prevention, resilience, and improved public health.

The studies assembled in this Research Topic reinforce the growing recognition that environmental and occupational exposures are not merely toxicologic phenomena, but complex biological, public health, economic, and societal challenges with consequences that may extend across multiple organ systems, populations, and generations (3, 4, 15, 29, 30). Overall, these investigations support a shift away from traditional paradigms toward integrated approaches that account for complex exposures, multisystem disease mechanisms, human factors, and systems-based prevention strategies (2, 17, 23). Like adverse events in healthcare, occupational exposures often emerge from latent organizational conditions, weak regulatory oversight, production pressures, communication failures, inadequate surveillance, and poorly designed work systems rather than isolated unsafe acts (Figure 1) (24, 25).

The importance of convergence science extends beyond scientific advancement. Coordinated efforts spanning epidemiology, toxicology, engineering, environmental science, medicine, and public policy have produced major improvements in global health, including reductions in tobacco-related disease, elimination of leaded gasoline, improvements in occupational safety, and mitigation of ozone depletion through the Montreal Protocol (3, 16, 29). Conversely, failures to recognize and respond to asbestos exposure, lead toxicity, environmental injustice, climate change, and military toxic exposures have resulted in preventable disease, disability, premature mortality, and substantial societal costs (3, 15, 22, 30).

Emerging digital technologies including wearable sensors, environmental monitoring networks, electronic health records, geospatial analytics, and artificial intelligence offer unprecedented opportunities for real-time exposure surveillance and earlier disease recognition, provided they are implemented within trustworthy governance frameworks (15, 31, 32). Invisible hazards demand visible systems. The future of environmental and occupational health will depend not only on discovering new toxicants but on redesigning the systems in which people live, work, and receive care (17, 23). Protecting future generations will require convergence not only of scientific disciplines but of policy, engineering, clinical medicine, occupational health, communities, and society itself (16, 29).

Environmental exposure science should evolve into a learning health system that continuously detects hazards, implements preventive interventions, evaluates outcomes, and redesigns policy. Integrating environmental monitoring, clinical outcomes, occupational surveillance, and policy feedback into continuously improving prevention strategies will help inspire, engage and support sustainable practices. The next frontier is not exposure science alone, but exposure intelligence: integrating surveillance, implementation science, systems engineering, and public policy to protect future generations.
